# Sintering Behavior of Spark Plasma Sintered SiC with Si-SiC Composite Nanoparticles Prepared by Thermal DC Plasma Process

**DOI:** 10.1186/s11671-017-2370-8

**Published:** 2017-11-25

**Authors:** Yeon-Tae Yu, Gautam Kumar Naik, Young-Bin Lim, Jeong-Mo Yoon

**Affiliations:** Division of Advanced Materials Engineering and Research Centre for Advanced Materials Development, Chonbuk National University, Jeonju, 54896 South Korea

**Keywords:** Si-SiC nanoparticles, Additive, Plasma processing, Spark plasma sintering

## Abstract

The Si-coated SiC (Si-SiC) composite nanoparticle was prepared by non-transferred arc thermal plasma processing of solid-state synthesized SiC powder and was used as a sintering additive for SiC ceramic formation. Sintered SiC pellet was prepared by spark plasma sintering (SPS) process, and the effect of nano-sized Si-SiC composite particles on the sintering behavior of micron-sized SiC powder was investigated. The mixing ratio of Si-SiC composite nanoparticle to micron-sized SiC was optimized to 10 wt%. Vicker’s hardness and relative density was increased with increasing sintering temperature and holding time. The relative density and Vicker’s hardness was further increased by reaction bonding using additional activated carbon to the mixture of micron-sized SiC and nano-sized Si-SiC. The maximum relative density (97.1%) and Vicker’s hardness (31.4 GPa) were recorded at 1800 °C sintering temperature for 1 min holding time, when 0.2 wt% additional activated carbon was added to the mixture of SiC/Si-SiC.

## Background

Silicon carbide (SiC) ceramics have been attracting great attention due to its phenomenal properties, such as high-temperature hardness, wear resistance, low thermal expansion coefficient, high thermal conductivity, strong corrosion resistance, and high stability in aggressive environment, and have been applied for various fields such as turbine blades, diesel engine parts, and aerospace and nuclear reactor materials [1–6]. However, it is difficult to densify the SiC without additives because of the covalent nature of Si–C bonding and low self-diffusion coefficient [7, 8]. The bulk SiC materials are usually prepared either by the solid state sintered silicon carbide (SSS-SiC) or by the liquid phase sintered silicon carbide (LPS-SiC) from the starting SiC crystalline powders [7, 8]. In the case of SSS-SiC, no liquid forming additives, such as boron, aluminum, carbon, or their compounds, have been used for densification of SiC by the reduction of the surface energy of grains and the reaction between silica present on surface and carbon. However, this process requires over 2000 °C temperature for sintering [7, 9, 10]. LPS-SiC is governed by liquid phase formation of metal oxide additive at sintering temperature and this liquid phase act as a mass transport media during SiC sintering [8, 11, 12]. Except magnesia and alumina, yttria and other rare earth oxides are mostly used as sintering additives, and sintering temperature can be decreased down to 1850 °C, depending upon the used combination of sintering additives [11, 12]. However, presence of the amorphous silicate compound at grain boundaries and the triple points cause decrease of hardness and high-temperature creep resistance as compared with the SSS-SiC [12]. But the additives used to enhance processing invariably become a “weak” secondary phase in the final ceramic, which usually lower its mechanical properties at high temperature [13]. This detrimental effect infers that the smallest fraction of additives is desirable. In addition, the effectiveness of the additives greatly depends on the homogeneity of their distribution [13]. There is also another approach for fabrication of bulk SiC, which is called as reaction bonded silicon carbide (RB-SiC). In RB-SiC, the reaction of molten silicon with carbon powder results in a formation of SiC [13–16]. Although this approach requires lower sintering temperature and there is no limitation of product shape and size, low density of the bodies is a disadvantage [17, 18]. However, lowering of sintering temperature is essential to save the energy. In recent time, energy saving becomes the driving force to find other methods suitable for the preparation of bulk SiC ceramics at low temperature.

Recently, nano-sized SiC has been widely investigated to examine their mechanical, physical, and chemical properties that are different from those in bulk forms and often useful [19–21]. For example, nanopowders primarily due to the higher specific surface areas and surface activities can provide the low-temperature sinterability of nano-sized SiC in the consolidation processing and the improvement of mechanical properties by making it possible to reach high densities [22]. Therefore, in present, we have developed a new method to prepare Si-coated SiC (Si-SiC) nanoparticle to apply as a sintering additive by using non-transferred thermal DC plasma processing of solid-state synthesized SiC powder [23].

In this study, the nano-sized Si-SiC composite particle as a sintering additive was applied for preparing bulk SiC ceramic by spark plasma sintering (SPS) process, and the effect of addition of the nano-sized Si-SiC composite particle on sintering temperature, relative density, and Vicker’s hardness of sintered SiC ceramic was investigated. In addition, to further increase the relative density and hardness of sintered SiC, reaction bonding between free silicon of nano-sized Si-SiC particle and activated carbon which was additionally added was newly introduced to SiC sintering process. The sintering mechanism of the SiC ceramic produced with nano-sized Si-SiC composite additive through SPS process was also discussed on the basis of nano-size effect and reaction bonding effect. This study provides a new promising strategy to be able to prepare the SiC ceramic with high density and hardness at a relatively low sintering temperature.

## Experimental

**Fig. 1:**
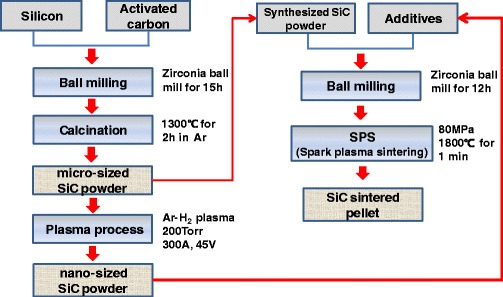
Experimental procedure for preparing micron-sized SiC powder by calcination and nano-sized Si-SiC composite powder by plasma and for sintering SiC ceramics by SPS

Figure 1 shows the preparation procedures of micron-sized SiC powder by solid-solid reaction (calcination), nano-sized SiC powder by non-transferred arc thermal plasma process, and sintered SiC pellet by SPS process. In this work, SiC powders with two different sizes, micron-sized SiC powder (as a main sintering material) and nano-sized Si-SiC composite nanopowder (as a sintering additive), were prepared by calcination and plasma processes respectively.

### Micron-sized SiC Powder Preparation

The micron-sized SiC was synthesized by using Si powders with an average particle size of 25 μm (99.9%; Neoplant Co. LTD.) and activated carbon with an average particle size of 32 μm (Sigma-Aldrich). In a typical procedure, 1:1.5 mol ratio of Si and carbon were mixed together by using ball mill for 15 h. The mixed powder was placed in a vertical tube furnace and heated at 1300 °C for 2 h with 10 °C/min heating rate in the presence of argon gas (1 L/min). After completion of the reaction, the obtained powder was grinded in agate mortar for further characterization.

### Plasma Processing of Synthesized SiC Powders

Plasma processing was carried out by non-transferred arc thermal plasma reactor as reported in our previous work [21, 23]. The milled SiC powder was fed into the plasma arc through the internal feeding pipeline of 2-mm inner diameter in the plasma torch using a specially designed powder feeder. The powder feeding system consisted of a sample container, a vibrator, and a carrier gas line. Powders were fed by vibrating feeder at 70 V with 1 g/min feeding rate. Typical synthesis experiments were operated at system pressures of 200 Torr, with Ar plasma gas flow rates of 30 L/min, H_2_ gas flow rates of about 3 L/min, and DC current of 300 A (at 45 V). After plasma ignition, a micron-sized SiC powder was supplied by feeder. The synthesized nanopowders were collected from the reactor wall and bottom of the plasma reactor system. The yield was about 80–85%.

### Preparation of Sintered SiC Pellet

Sintered SiC pellet was prepared by SPS process (as shown in Fig. 1). Both SiC materials, i.e., the micron-sized SiC powder synthesized by calcination process and the nano-sized Si-SiC powder obtained from plasma process, were used without additional additives. The mixing content of Si-SiC nanoparticles in micron-sized SiC powder was changed from 5 to 15 wt%.

The mixed powders were put into a graphite die (20 mm in diameter) and sintered with SPS system in vacuum atmosphere (10^−2^ Torr). The heating rate was fixed at 600 °C/min, and the applied pressure was 80 MPa. The sintering temperature was changed from 1600 to 1800 °C. The holding time at target temperature was varied from 0 to 1 min at 1800 °C. After sintering, the sample surfaces were grounded to remove the graphite layer and then polished by a diamond paste. The density of the sintered specimens was measured by the Archimedes method in deionized water as an immersion medium.

### Sample Characterization

The crystallographic structures of the solid samples were determined using a XRD (D/Max 2005 Rigaku) equipped with graphite monochromatized high-intensity Cu-Kα1 radiation (*λ* = 1.5405 Å). The XRD patterns were recorded from 20° to 80° (2*θ*) with a scanning speed of 0.04°/s. Particle size and morphology were investigated by a scanning electron microscope (SEM; JSM-5900, JEOL) and transmission electron microscope (TEM; JEM-2010, JEOL).

## Results and Discussion

**Fig. 2:**
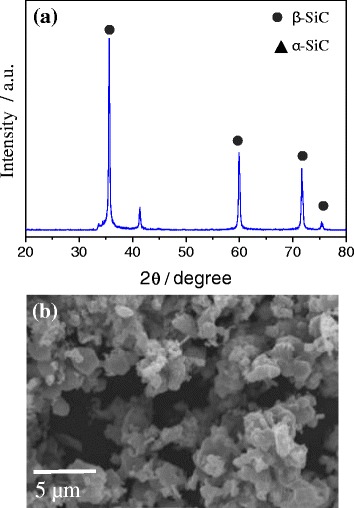
XRD profile (**a**) and FESEM image (**b**) of micron-sized SiC synthesized by solid-state method

Figure 2 shows the XRD pattern and FESEM image of SiC synthesized by a solid-state method using mixture of Si and C in 1:1.5 mol ratios. XRD pattern confirms the formation of β-SiC with small amount of α-SiC as shown in Fig. 2a. There were no other impurity phases, such as free silicon and SiO_2_. FESEM image showed the formation of micron-sized SiC particles and the particle size varied from 2 to 5 μm as displayed in Fig. 2b.

**Fig. 3:**
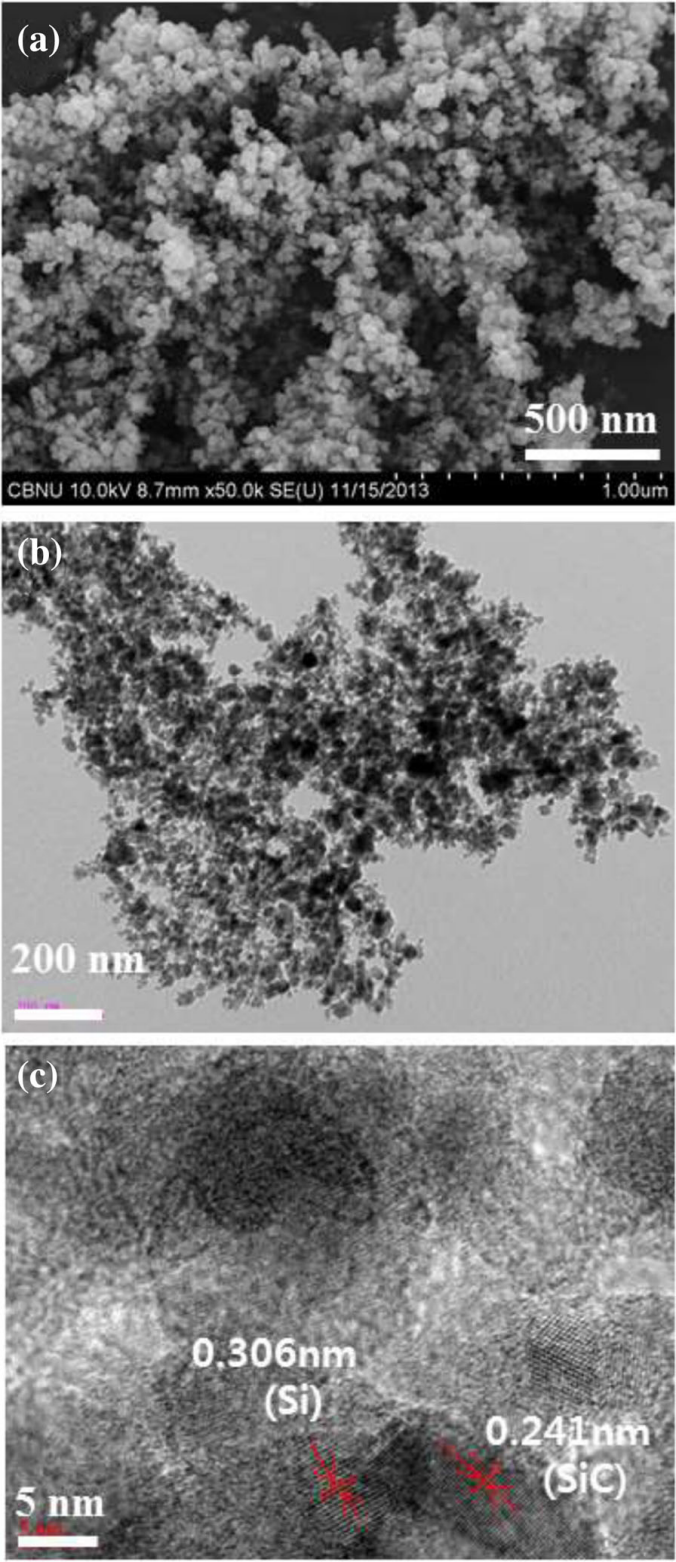
EM images of Si-SiC composite particles prepared by non-transferred arc thermal plasma process: **a** FESEM, **b** TEM, and **c** HR-TEM

Nano-sized SiC powder was prepared from this micron-sized SiC powder using thermal plasma processing as exhibited in Fig. 3. Figure 3a, b was a FESEM image and a TEM image, respectively. These photographs confirm the formation of nano-sized Si-SiC particles, and particle size varied from 20 to 70 nm. Figure 3c is a HRTEM image of Si nano-sized SiC particles, which confirms the formation of nano-sized Si-SiC composite particles as clear lattice fringes of both materials (Si and SiC) are presented. The surface area of nano-sized SiC powder was 69 m^2^/g.

**Fig. 4:**
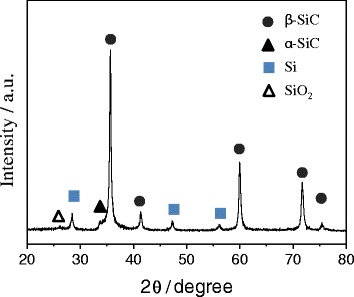
XRD profile of Si-SiC composite nanoparticles prepared by non-transferred arc thermal plasma process

The phase and structure of nano-sized SiC particles were analyzed by XRD and shown in Fig. 4. Similar to micron-sized SiC, it also shows the formation of β-SiC with small amount of α-SiC. However, nano-sized SiC exhibited free Si and SiO_2_ peaks. The appearance of silicon peak was related to the partial decomposition of SiC during thermal plasma processing. The origin of SiO_2_ peak was possibly related to the partial oxidation of SiC surface during the exposure to air after plasma processing. Sintered SiC ceramic pellet was prepared from these two kinds of SiCs, i.e., the micron-sized SiC and the nano-sized Si-SiC composites. The mixture was sintered by SPS process using various compositions of micron-sized SiC and nano-sized Si-SiC, and sintering temperature, holding time at sintering temperature, and compressed pressure were varied as shown in Table 1.

**Table 1:** Composition of micron-sized SiC and nano-sized Si-SiC composite particle, temperature, and pressure for preparing sintered SiC pellets and their relative densities and Vicker’s hardness

Sample	Composition (wt%)		Temp. (°C)	Holding time (min)	Pressure (MPa)	Relative density (%)	Vicker’s hardness (GPa)
	Micron-sized SiC	Nano-sized Si-SiC					
a	90	10	1600	0	80	78.7	9.8
b	90	10	1700	0	80	86.1	14.8
c	90	10	1800	0	80	87.4	18.6
d	90	10	1800	1	80	88.2	21.2

The changes of relative density and Vicker’s hardness of the sintered SiC according to sintering temperature and holding time at target sintering temperature are also given in Table 1. Relative density and hardness increases with increasing sintering temperature, and highest relative density (87.4%) and hardness (18.6 GPa) were recorded at 1800 °C. The relative density and hardness was further increased to 88.2% and 21.2 GPa, respectively, with increasing holding time from 0 to 1 min at 1800 °C sintering temperature. It suggest that relative density and hardness increases with increasing holding time; unfortunately, the holding time at 1800 °C could not be increased further due to limitation of the SPS system.

**Fig. 5:**
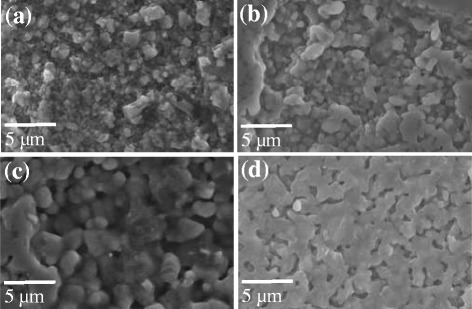
FESEM images of the sintered SiC with nano-sized Si-SiC composite particles by SPS process at **a** 1600 °C, **b** 1700 °C, **c** 1800 °C sintering temperature with 0 min holding time, and **d** 1800 °C sintering temperature at 1 min holding time at target temperature

Figure 5 shows the FESEM images of sintered SiC surface with varying sintering temperature and holding time. The grain size of SiC was increased with increasing sintering temperature as shown in Fig. 5a–c. The shape and size of micron-sized SiC particles were almost maintained up to 1600 °C (Fig. 5a), and the grain growth of SiC was started from 1700 °C which resulted in an increase in relative density and hardness of up to 86.1% and 14.8 GPa, respectively (Fig. 5b). At 1800 °C of sintering temperature, the grain size of SiC was 2–4 μm and the crystal texture was more densified (Fig. 4c). The relative density and hardness at 1800 °C was 87.4% and 18.6 GPa, respectively. Grain growth was further recorded when sample was held for 1 min at 1800 °C sintering temperature, which is evident from the increase in relative density and hardness to 88.2% and 21.2 GPa, respectively (Fig. 5d).

In order to increase the relative density, the adding amount of nano-sized Si-SiC in micron-sized SiC powder was changed from 5 to 15 wt%. In addition, extra activated carbon was also added to this mixture to increase the relative density through reaction bonding (RB) with free silicon of nano-sized Si-SiC. The relative density and Vicker’s hardness of sintered SiC depending on different compositions of micron-sized SiC, nano-sized Si-SiC, and activated carbon is summarized in Table 2.

**Table 2:** Composition of micron-sized SiC, nano-sized Si-SiC composite particle, and activated carbon for preparing sintered SiC pellets, and their relative densities and Vicker’s hardness (sintering temp. 1800 °C, holding time 1 min, pressure 80 MPa)

Sample	Composition (wt%)			Relative density (%)	Vicker’s hardness (GPa)
	Micron-sized SiC	Nano-sized SiC	Activated carbon		
a	95	5		85.9	21.1
b	90	10		88.2	21.2
c	85	15		85.6	16.6
d	89.9	10	0.1	93.1	25.2
e	88.8	10	0.2	97.1	31.4

**Fig. 6:**
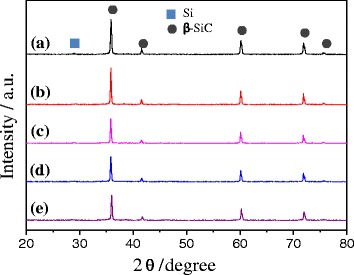
XRD profiles of SiC pellets sintered with nano-sized Si-SiC composite particles and activated carbon by SPS process (details of a, b, c, d, and e are given in Table 2)

Sintering temperature (1800 °C), holding time (1 min), and pressure (80 MPa) were kept constant throughout the experiment. In the absence of added carbon, the relative density and hardness increases with increasing nano-sized SiC content up to 10 wt% and then decreases. For example, the relative density and hardness was 85.9% and 21.1 GPa, when the content of nano-sized Si-SiC was 5 wt%. The relative density and hardness increased to 88.2% and 21.2 GPa, respective at 10 wt% of the nano-sized Si-SiC. Whereas when the content was 15 wt%, the hardness decreased remarkably down to 16.6 GPa though the relative density showed still 85.6%. This is mainly because the excessively added nano-sized Si-SiC particles can provide large amount of pore in the sintered SiC. For further increase of relative density and hardness, activated carbon was added additionally. The addition of 0.1 wt% of activated carbon resulted in remarkable increase in the relative density (93.1%) and hardness (25.2 GPa). The relative density and hardness was further increased to 97.1% and 31.4 GPa, respectively, with increasing activated carbon content up to 0.2 wt%. XRD analysis of these sintered SiC pellets with different compositions was carried out and shown in Fig. 6. There was no change in crystal structure even after sintering, except for a weak silicon peak recorded at 29°, which is possibly originated from the addition of nano-sized Si-SiC composite particles.

**Fig. 7:**
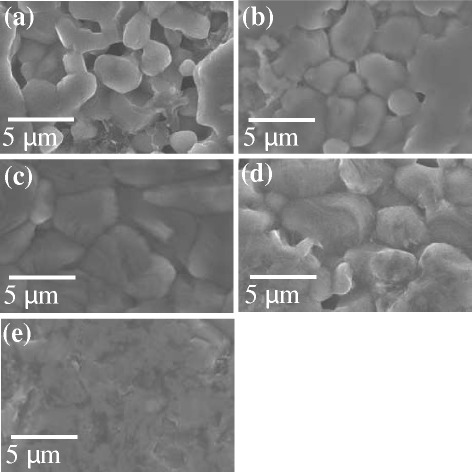
FESEM images of SiC pellets sintered with nanosized Si-SiC composite nanoparticles and activated carbon by SPS process (details of **a**, **b**, **c**, **d**, and **e** are given in Table 2)

Figure 7 shows FESEM images of the surface of the sintered SiC pellets with different micron-sized SiC and nano-sized Si-SiC compositions. The grain size of SiC was 2–3 μm with bigger pores, when nano-sized Si-SiC content was 5 wt% (Fig. 7a). The grain size was increased with increasing the content of nano-sized Si-SiC as displayed in Fig. 7b, c. The grain size of SiC pellets with 10 and 15 wt% of Si-SiC was about 3–5 and 4–6 μm, respectively. Figure 7d, e presents the FESEM images of sintered SiC pellet after addition of activated carbon. When 0.1 wt% of activated carbon was added, giant grains start to appear, which suggest that densification of SiC texture was progressed by reaction bonding between silicon and activated carbon. Further increase in activated carbon up to 0.2 wt% resulted in complete densification of SiC texture as shown in Fig. 7e.

**Fig. 8:**
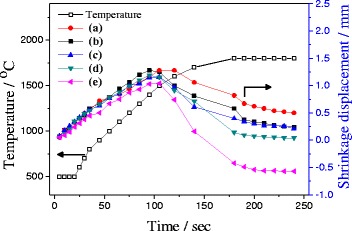
Shrinkage displacement change of SiC pellets sintered with nanosized Si-SiC composite nanoparticles during SPS process (details of a, b, c, d, and e are given in Table 2)

Figure 8 presents the shrinkage displacements of sintered SiC samples with different compositions at different sintering temperature. There was an expansion recorded for all specimens up to 1500 °C, which is mainly because of expansion of gases present in SiC mixture (Fig. 8a–e). It can also be observed by 1.0–1.28 mm increase in height of graphite mold having SiC mixture powder after sintering as compared to initial sintering step. Further increase in sintering temperature resulted in shrinkage of all specimens due to sintering effect of nano-sized Si-SiC nanoparticles. The increase in holding time of specimen at sintering temperature from 0 to 1 min also resulted in increase in shrinkage of specimens. The addition of extra activated carbon to the mixture of SiC and Si-SiC showed higher shrinkage displacement after 1500 °C as compared to SiC/Si-SiC mixtures without activated carbon (Fig. 8d, e). Furthermore, the shrinkage displacement increases with increasing added amount of additional activated carbon. For example, shrinkage displacement was increased from 1.11 to 1.61 mm, when added activated carbon amount was increased from 0.1 to 0.2 wt%, respectively, as displayed in Fig. 8d, e. This is due to the reaction bonding effect of free silicon of Si-SiC composite nanoparticle and activated carbon. The exothermic nature of this reaction results in increase in real temperature inside SiC pellet during sintering. Thus, these results clearly confirm that reaction bonding effect of silicon and activated carbon can increase the relative density and hardness of SiC ceramics.

**Fig. 9:**
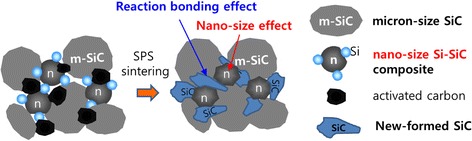
Schematic drawing of sintering mechanism for micron-sized SiC powder with nano-sized Si-SiC composite powder and activated carbon as sintering additives

From the above experimental results, we can summarize a sintering mechanism of micron-sized SiC powder with nano-sized Si-SiC composite powder and activated carbon as follows. It was determined in Table 1 that the sintering of micron-sized SiC powder with 10 wt% of nano-sized Si-SiC composite powder was started at 1600 °C, and the sintering reaction was accelerated with increasing the sintering temperature and holding time. From this result, we confirmed the nano-size effect of Si-SiC composite nanoparticles on the sintering of the micron-sized SiC powder. Herein, if activated carbon is added to the mixture of micron-sized SiC and nano-sized Si-SiC composite powder, the reaction bonding, which originated from the exothermic reaction between the free silicon of Si-SiC composite nanoparticles, can be induced into the sintering process as shown schematically in Fig. 9. Consequently, it could be found that the sintering mechanism of micron-sized SiC powder with nano-sized Si-SiC composite powder and activated carbon as sintering additives lies on both effects, the nano-size effect and the reaction bonding effect, which were introduced from the nano-sized Si-SiC composite powder.

## Conclusions

Micron-sized SiC (2–5 μm) powder was synthesized by a solid-state method using Si powder and activated carbon sources. Nano-sized Si-SiC composite powder, having 20–70 nm particle size, was prepared by non-transferred arc thermal plasma process. Sintered SiC pellets were prepared by SPS process using the mixture with different ratio of micron-sized SiC powder and nano-sized Si-SiC composite particle as a sintering additive. At a fixed ratio of micron-sized SiC and nano-sized Si-SiC (90:10), the relative density and Vicker’s hardness increased with increasing sintering temperature and holding time. The maximum relative density (88.2%) and Vicker’s hardness (21.2) were recorded at 1800 °C sintering temperature for 1 min holding time. The relative density and Vicker’s hardness was further increased by addition of extra activated carbon to the mixture of micron-sized SiC and nano-sized Si-SiC. The relative density and Vicker’s hardness were increased to 97.1% and 31.4 GPa, respectively, with the addition of 0.2 wt% of extra activated carbon to the SiC/Si-SiC mixture. It was found that the nano-size effect of Si-SiC composite particle and the exothermic nature of silicon–carbon reaction bonding were responsible for the increase in relative density and hardness. Therefore, it was suggested that the nano-sized Si-SiC composite particle could be a promising additive for sintering of SiC ceramics.
